# Plasma Interleukin-18 and Dendritic Cells in Males with Psoriasis Vulgaris

**DOI:** 10.1155/2007/61254

**Published:** 2007-04-10

**Authors:** Aldona Pietrzak, Konrad Janowski, Grażyna Chodorowska, Anna Michalak-Stoma, Jacek Roliński, Anna Zalewska, Iwona Jastrzębska, Jacek Tabarkiewicz, Tomasz Paszkowski, Ewa Kapeć, Dorota Krasowska

**Affiliations:** ^1^Chair and Department of Dermatology, Venerology and Paediatric Dermatology, Medical University of Lublin, 13 Radziwiłłowska Street, 20-080 Lublin, Poland; ^2^Department of Adult Clinical Psychology, John Paul II Catholic University of Lublin, 14 Racławickie Avenue, 20-950 Lublin, Poland; ^3^Department of Clinical Immunology, Medical University of Lublin, 8 Jaczewskiego Street, 20-090 Lublin, Poland; ^4^Department of Dermatology, Medical University of Łódź, 5 Krzemieniecka Street, 94-017 Łódź, Poland; ^5^3rd Department of Gynaecology, Medical University of Lublin, 8 Jaczewskiego Street, 20-090 Lublin, Poland

## Abstract

Peripheral blood dendritic cells seem to play a crucial role in psoriatic inflammatory processes. The aim of our study is to investigate the relationship between plasma interleukin-18 (IL-18) levels and blood dendritic cells in psoriatic patients. IL-18 plasma levels were measured by ELISA. Phenotypes of dendritic cell subsets were analyzed by double-colour flow cytometry. Plasma IL-18 level in psoriatic males was significantly higher, whereas counts of BDCA-2+ cells were lower than in the control group. The myeloid/plasmacytoid ratio was significantly higher in the patient group compared to the control one. In the patient group, significant negative correlations between plasma IL-18 level and both the BDCA-1+ and BDCA-2+ counts were found. BDCA-1+ counts correlated negatively with percentage of skin involvement. IL-18 seems to play a role in psoriasis pathogenesis. The decreased counts of blood plasmacytoid DCs in psoriatic patients might result from IL-18 down-regulation of plasmacytoid DC precursor proliferation.

## 1. INTRODUCTION

Interleukin-18 (IL-18) is produced by a wide range of cells, primarily macrophages, monocytes, and to a smaller degree by keratinocytes and osteoblasts [1]. It strengthens cytotoxic properties of natural killer (NK) cells and CD8+ T cells. It also possesses a unique ability to increase the activity of both Th1-type as well as Th2-type CD4+ T cells and depends on local cytokine network [2]. IL-18 contributes to inflammation and fever due to its potent inducing effects on the gene expression and synthesis of interferon-*γ* (IFN-*γ*), granulocyte-macrophage colony-stimulating factor (GM-CSF), tumour necrosis factor (TNF), interleukin-1 (IL-1), fas ligand (FasL), and several chemokines [3–7]. Being a pleiotropic cytokine, IL-18 can play an immunoregulatory role, especially in inflammatory, infectious, and autoimmune diseases.

Only a few studies analysed the role of IL-18 in psoriasis vulgaris [4, 8–10], they did not, however, yield conclusive results [11]. Increased expression of IL-18 was demonstrated in keratinocytes from psoriatic lesions in comparison to keratinocytes from normal skin [12–15]. However, neither the bioactivity of IL-18 nor the influence of IFN-*γ* on inflammatory processes in the peripheral blood mononuclear cells (PBMC) from psoriatic scales could be detected [12]. The level of IL-18 mRNA was demonstrated to be 2- to 8-fold higher in extracts from psoriatic skin lesions than the one obtained from both uninvolved skin extracts in psoriasis patients and healthy subjects. Similarly, 6- to 8-fold higher concentrations of IL-18 receptors mRNA were observed in psoriatic lesional skin in comparison to both uninvolved skin of psoriasis patients and healthy subjects [13–15].

In the human peripheral blood, two distinct lineages of dendritic cells (DCs) were identified—myeloid and plasmacytoid dendritic cells (mDCs and pDCs, resp.) [16]. These two subsets of DCs have been implicated in inducing diverse types of immune responses, with mDCs favouring Th1- and pDCs favouring Th2-type immune reactions profile [17]. These two DCs subsets can be distinguished by their different surface antigens. mDCs express specific antigen-blood dendritic cell antigen BDCA-1, whereas pDCs express characteristic antigen BDCA-2 [18].

Recent data indicate that DCs may be involved in pathological processes in numerous diseases, including immune-mediated skin conditions [19, 20]. In psoriasis, BDCA-2 cells were detected in the basal layer of the epidermis and the papillary dermis [20]. This observation is particularly interesting in the context of the most important immunopathogenic hypotheses of psoriasis focused on the synthesis and release of proinflammatory mediators by activated DCs and T cells, distorted function of the immune synapse leading to the emergence of self-reactive T cells or epidermal expression of chemokines attracting T cells and DCs into the skin [21].

Some studies suggested that DCs may be associated with IL-18. Gutzmer et al. [22] have recently demonstrated that human DCs express IL-18 receptors and that IL-18 has a direct chemotactic effect on DCs. Increased IL-18 levels, observed in some diseases, might lead to DCs recruitment to the sites of inflammation. Companjen et al. [11] found that in the epidermis, IL-18 expression was detected not only in keratinocytes but also in the cells with a dendritic morphology, probably Langerhans cells. The cellular infiltrate in the dermal papillae of psoriatic lesional skin, containing cells with dendritic and lymphocyte morphology, also expressed IL-18.

Since several lines of evidence link the pathogenesis of psoriasis to both increased levels of IL-18 and DCs functions, we decided to investigate the relationship between plasma IL-18 and blood DCs in psoriasis patients. We hypothesized that such correlation may help to explain some of the pathogenic processes observed in the psoriatic skin.

## 2. MATERIALS AND METHODS

### 2.1. Participants

Thirty-eight males with psoriasis and 21 healthy male volunteers took part in the study. Patients were hospitalized at Department of Dermatology, Venerology and Paediatric Dermatology, Medical University of Lublin, Poland, due to psoriasis vulgaris. Patients had a chronic active type of psoriasis of moderate severity with the mean psoriasis area and severity index (PASI) of 24.56 ± 8.28 (M ± SD) and the average percentage of skin involvement of 28.44 ± 19.54%. The severity of psoriasis was evaluated in each patient by the same investigator. The mean duration of psoriasis calculated in months was 152.10 ± 149.46. The patients treated with topical or systemic steroids, any form of dithranol or retinoid therapy prior to examination, were excluded from entering the study. Subjects with other systemic, infectious, parasitic, neurological, or psychiatric diseases were also excluded from the study. The blood samples were collected from all the participants in the morning. The patients remained seated for at least 15 minutes prior to blood collection. An informed written consent was obtained after complete description of the study from each participant. The study was approved by the Ethics Committee of the Medical University of Lublin.

### 2.2. Evaluation of DCs subpopulations

**Figure 1:**
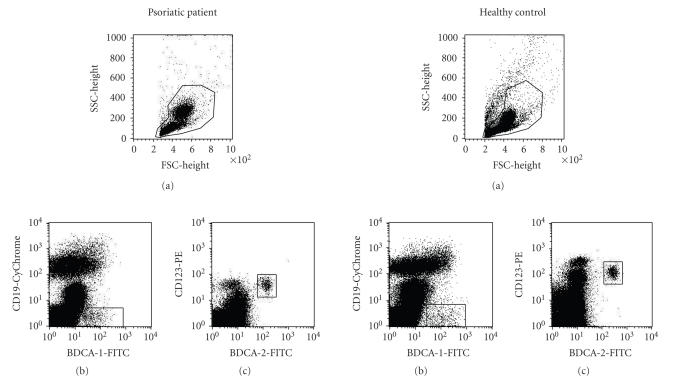
(a) Myeloid and plasmacytoid DCs were quantified as the percentages of peripheral blood mononuclear cells, (b) mDCs were defined as BDCA-1 positive and simultaneously CD19 negative cells, (c) pDCs as double BDCA-2 and CD123 positive cells. Correlations between IL-18 and DCs in patients with psoriasis and healthy controls.

The mononuclear cells were isolated from heparinised blood by density gradient centrifugation on Lymphoprep (Nycomed, Norway) and washed twice in phosphate buffered saline without Ca^2+^ and Mg^2+^ containing 0.5% bovine serum albumin and 2 mM EDTA. The phenotypes of DCs were analysed with double-colour flow cytometry using monoclonal antibodies: mouse antihuman BDCA-1-FITC (Miltenyi-Biotec, Germany), CD19-CyChrome (Pharmingen, USA), BDCA-2-FITC (Miltenyi-Biotec, Germany), CD123-PE (Becton Dickinson, USA), and matched isotype control (Caltag, USA). The cell staining was performed according to the manufacturers' instructions in the presence of FcR blocking reagent (Miltenyi-Biotec, Germany). The immunolabelled cells were collected (a total of 300 000 events) using an FACSCalibur flow cytometer equipped with 488 nm argon laser (Becton Dickinson) and analysed with cell-quest software. The numbers of myeloid and plasmacytoid DCs were quantified as the percentages of peripheral blood mononuclear cells. mDCs were defined as BDCA-1 positive and simultaneously CD19 negative cells, whereas pDCs as double BDCA-2 and CD123 positive cells (Figure 1).

### 2.3. Evaluation of IL-18 plasma concentration

Plasma IL-18 concentration was determined by a commercially available Human IL-18 ELISA kit (MBL, Japan). Plasma samples were collected on EDTA as anticoagulant and stored deep frozen at −80°C until further evaluated. The analytical procedure was performed according to the manufacturers' instructions. ELISA Reader Elx800 (BIO-TECK Instruments) was used for the examination. The detection limit of Human IL-18 ELISA kit is 12.5 pg/mL.

## 3. STATISTICAL ANALYSIS

Results are presented as mean, median, 25 percentile and 75 percentile, mean (M), standard deviation (SD). The fit of the data to the normal distribution was tested with Kolmogorov-Smirnov test. Since the distribution of the data was significantly different from normal, nonparametric statistics were further used. Mann-Whitney *U* test was employed for comparison between patient and control groups, and Spearman *rho* correlations coefficients were calculated between the assessed variables. A *P* value <.05 was considered statistically significant.

## 4. RESULTS

### 4.1. Group characteristics

The patients and healthy controls were homogeneous with respect to the number of demographic, physical, and behavioural variables including age, weight, height, body mass index (BMI), blood pressure, WBC, frequency of smoking, and declared alcohol intake (Table 1). None of the differences between the patients and healthy subjects in the mean values of these variables proved to be statistically significant (*P* < .05).

### 4.2. IL-18 levels and DCs counts in patients and healthy subjects

**Figure 2:**
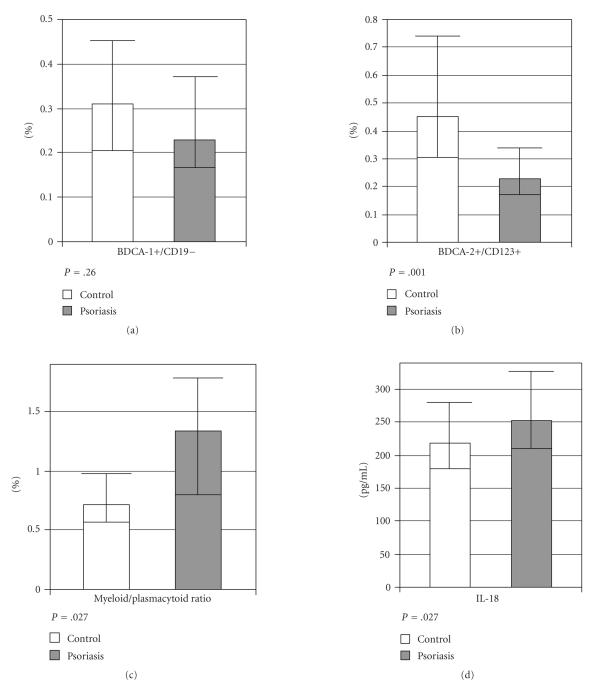
Comparison between men with psoriasis and healthy subjects: (a) percentages myeloid BDCA-1+/CD19- DCs, (b) plasmacytoid BDCA2+/CD123+ DCs, (c) myeloid/plasmacytoid ratio, and (d) blood concentration of IL-18. Results are shown as median values and 25–75 percentiles.

The mean plasma level of IL-18 was significantly increased in males with psoriasis as compared to the healthy control male group (*P* = .027). The counts of BDCA-1+ cells in psoriasis patients were not significantly different from those in healthy controls (*P* < .05). The counts of BDCA-2+ cells were found to be significantly lower in patients with psoriasis than those in healthy controls (*P* = .001). The myeloid/plasmacytoid ratio was significantly higher in psoriatic patients compared to the control group (Figure 2).

### 4.3. Correlations between IL-18 and DCs in patients and healthy subjects

**Figure 3:**
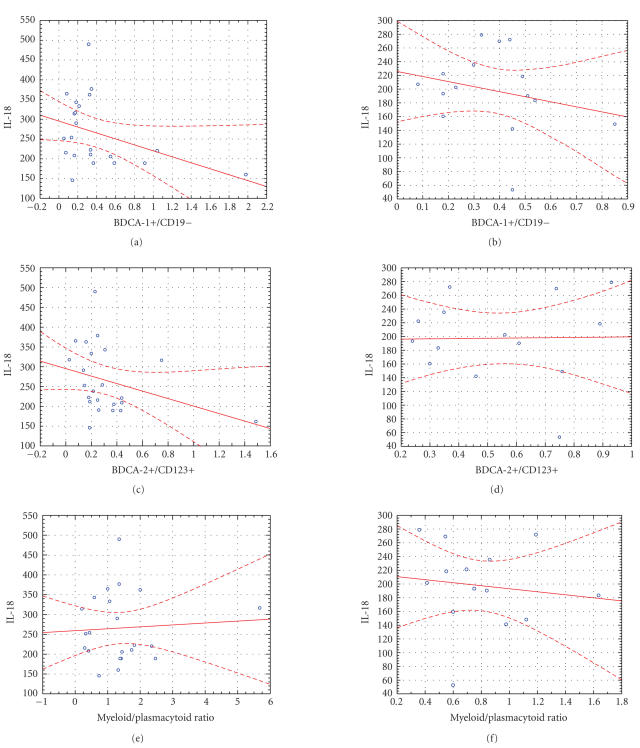
Correlations between IL-18 and DCs in patients with psoriasis and healthy controls.

In the correlations between IL-18 and BDCA-1+ cells, BDCA-2+ cells were examined separately for the patient and the control groups. In the latter, none of the correlation coefficients was found to be statistically significant. In the patient group, however, statistically significant negative correlations were found both between the BDCA-1+ cell counts and plasma IL-18 level (*rho* = −0.38) as well as the BDCA-2+ cell counts and plasma IL-18 level (*rho* = −0.41) (Table 2, Figure 3).

### 4.4. Correlations between IL-18, DCs, and clinical severity of psoriasis patients

In the patient group, correlation coefficients were calculated between the DCs subpopulations, IL-18, and clinical severity of the disease. Neither DCs nor IL-18 showed statistically significant correlations with the severity of psoriasis expressed by PASI score. IL-18 and BDCA-2+ did not demonstrate statistically significant correlations with percentage of skin involvement in psoriatic patients either. However, the severity of the disease, expressed by percentage of skin involvement correlated negatively with BDCA-1+ cell counts (*rho* = −0.39) (Table 3).

## 5. DISCUSSION

Our patients with psoriasis showed significantly increased levels of plasma IL-18 as compared to healthy subjects, which confirms findings reported by other authors [4], and our previous ones [8–10]. Increased IL-18 levels found in our patients seem to be in agreement with the widely accepted observation that IL-18 is a potent inducer of IFN-*γ* from T cells, NK cells, B cells, dendritic cells and, generally, is a trigger of the Th1 immune response [11, 23]. Taking into account the fact that psoriasis is a Th1-type disease, IL-18 may be regarded to be of some importance in its pathogenesis. This is further supported by studies demonstrating decrease of IL-18 levels after narrowband UVB therapy together with other parameters' characteristics for Th1 response [24]. Apart from stimulating the Th1 response, IL-18 can regulate the Th2 response depending on local cytokine network. IL-12 enhances IFN-*γ* production induced by IL-18, whereas IL-18 alone induces IL-4 and IL-13 production [23, 25].

These unique properties of IL-18 could explain statistically significant negative correlations between the BDCA-1+ cell counts and plasma IL-18 level as well as the BDCA-2+ cell counts and plasma IL-18 level. Kaser et al. [26] showed that plasmacytoid DC lineage express an *α* chain of IL-18 receptor (IL-18R*α*). Therefore, IL-18 can directly down-regulate proliferation of plasmacytoid DC precursors (pre-DC2s). This, in turn, can lead to a decrease in BDCA-2+ cells. IFN-*γ* was demonstrated to up-regulate the IL-18R on monocyte-derived dendritic cells [22, 27]. It might have the similar effect on dendritic cells in vivo. Our results show the statistically significant decrease of BDCA-2+ cells and the significantly higher myeloid/plasmacytoid ratio in the psoriatic patients compared to the control group. Spearman's rank correlation coefficients indicated that negative correlation between IL-18 and BDCA-2+ is more pronounced (Figure 3). This finding could be explained by both the direct influence of IL-18 on plasmacytoid DCs, which in psoriasis is enhanced by increased level of IL-12 [24], as well as additional function of IL-4. The Th2-type cytokine-IL-4 is able to induce apoptosis of plasmacytoid DCs, but IFN-*γ* can protect BDCA-2+ cells from this process [28].

Nestle et al. [29] observed a lower level of BDCA-2+ cells in the blood of psoriatic patients than in healthy subjects. They suggested that BDCA-2+ cells are decreased in the blood of psoriatic patients due to their migration into the lesional skin, a phenomenon similar to that observed in systemic lupus erythematosus. Therefore, the decreased counts of BDCA-2+ cells we found in our patients might possibly be related to their presumed migration into the lesional skin. This remains in agreement with the findings of other authors who observed increased DCs counts in the lesional psoriatic skin [20, 29].

Nestle et al. [29] found an elevated level of plasmacytoid DCs in both plaque lesions and uninvolved skin of psoriatic patients as compared to the skin of control subjects. Kaser et al. [26] analysed the role of IL-18 in the initial phase of Th response and demonstrated that IL-18 interacts with the differentiation pathway of plasmacytoid DCs. Through this modulation, IL-18 shifts the Th1/Th2 balance towards Th1 response. The potent chemotactic activity of IL-18 on plasmacytoid DCs was also identified. Further studies are, however, required to examine the chemotactic effect of IL-18 on plasmacytoid DCs in the psoriatic patients' skin and blood. What is more is that investigation on the expression of IL-18R on plasmacytoid DCs in the patients' skin and blood seems to be of considerable usefulness.

The counts of BDCA-1+ cells were not significantly different in the blood of our psoriatic patients from that of healthy controls. However, our study demonstrated a significant negative correlation (*rho* = −0.38) between plasma IL-18 and BDCA-1+ cell counts in patients with psoriasis. This negative correlation may be the result of myeloid dendritic cell downregulation driven by IFN-*γ*, as it was already demonstrated in systemic lupus erythematosus [30]. In conclusion, based on the increased IL-18 levels in plasma of our patients, this cytokine seems to play a role in the etiopathogenesis of psoriasis which was already suggested by many authors. Decreased blood plasmacytoid DCs in psoriatic patients might be related to the IL-18 down-regulation of plasmacytoid DC precursor proliferation. IFN-*γ*, by exerting its influence on myeloid/plasmacytid ratio, on the one hand could down-regulate myeloid dendritic cells and on the other hand can protect plasmacytoid dendritic cells from apoptosis. Explanation of the exact mechanisms of interactions between IL-18 and DCs in psoriasis requires, however, further studies.

## Figures and Tables

**Table 1:** Demographic, physical, and behavioural characteristics of patients with psoriasis and healthy controls.

	Patients				Healthy controls				Mann-Whitney *U* test	

	Mean	Median	25 percentile	75 percentile	Mean	Median	25 percentile	75 percentile	*Z*	*P*

Age (years)	37.74	41.0	25.00	47.00	34.04	33.5	24.00	39.50	1.20	.22
Weight (kg)	78.12	77.0	68.00	86.00	78.47	77.0	71.50	86.50	−0.40	.68
Height (cm)	174.64	177.0	171.00	180.00	176.5	176.5	173.00	181.00	−0.84	.40
BMI (kg/m^2^)	25.60	25.24	22.22	27.45	25.38	25.06	23.27	28.00	0.05	.95
RR1 (mmHg)	129.58	120.0	120.00	140.00	125.7	125.0	120.00	132.50	0.66	.50
RR2 (mmHg)	83.51	80.0	75.00	90.00	82.54	80.0	80.00	85.00	0.24	.80
WBC (K/*μ*L)	6.60	6.12	5.45	7.79	6.27	6.09	5.36	9.1	0.83	.40
Smoking (number/day)	5.8	0	0	10.00	7.12	1.5	0.00	15.00	−0.44	.65
Alcohol consumption (L/month)	0.61	0	0	0.50	0.74	0.25	0.10	0.70	−1.55	.12

**Table 2:** Spearman *rho* correlation coefficients between IL-18 and DCs in patients with psoriasis and healthy controls. Correlations marked by asterisk are statistically significant with *P* < .05.

		IL-18	
		
		Patients	Controls

BDCA-1+/CD19-	*rho*	−0.38*	−0.33
BDCA-2+/CD123+	*rho*	−0.41*	0.07
Myeloid/plasmacytoid ratio	*rho*	−0.008	−0.31

**Table 3:** Spearman *rho* correlation coefficients between IL-18, DCs, and clinical characteristics of psoriasis in patients with psoriasis. Correlations marked by asterisk are statistically significant with *P* < .05.

		PASI	Lesional skin (%)

IL-18	*rho*	0.071	0.26
BDCA-1+/CD19-	*rho*	−0.17	−0.39*
BDCA-2+/CD123+	*rho*	−0.07	−0.02
Myeloid/plasmacytoid ratio	*rho*	−0.16	−0.38*
